# Inflammation of the Brain

**Published:** 1847-08

**Authors:** G. N. Fitch

**Affiliations:** Professor of Institutes and Practice in Rush Medical College, Chicago


ARTICLE IV.

*Inflammation of the Brain.* By G. N. Fitch, M.D., Professor of Institutes and Practice in Rush Medical College, Chicago.

Among not the least of the difficulties which our profession encounters, is the variation of disease of the same organ in different latitudes, and under the different circumstances of age, constitution, previous habits and the thousand inscrutable ever-varying causes to which we are subjected. These variations form exceptions to any rule, and are often more numerous than the cases under the rule itself. Hence the necessity, if possible, of becoming familiar with all. Many of the exceptions of general occurrence are well known, and so well described in books of practice that a mistake in diagnosis can rarely occur; while others, either from being confined to certain localities or from being less defined in their nature and with symptoms more obscure, escape the observation of standard authors and are left to be detected as best they may be, if at all, by those under whose care they may fall. Such detection, in some cases, and among others in the case of the disease under consideration as it is found here, so far from being aided by the descriptions of standard medical authors, is greatly embarrarsed inasmuch as they enumerate groups of symptoms as always present and as necessary to constitute the disease, but one or two of which are ever found. The presumption, therefore, upon the part of the practitioner, naturally is, that he is dealing with an anomalous disease, instead of one in the treatment of which he would meet with but little embarrassment, did its written history enable him to diagnosticate it. That the disease in question, in the same form under which it not unfrequently prevails here to considerable extent, is not found elsewhere, is scarcely credible. Yet its prevalence to whatever extent in other localities in no manner benefits the profession here so long as the principal source from whence its junior members must derive their information, the books, lend them none but doubtful assistance.

Under the head of "*Inflammation of the Brain*," we include inflammation both of the substance and meninges, making no effort to distinguish between them. Indeed, such effort, even if it were successful, would be attended with no practical benefit, as the treatment, with our present knowledge, would be the same in both cases

We will first briefly examine its *Diagnosis* as given by some of our standard writers when we shall have the advantage of being able to compare the different descriptions with each other and with the disease as it presents itself at the bedside. Several of these descriptions will individually rather embarrass than aid one in his diagnosis, but all collectively may assist in arriving at some conclusion relative to the absence or presence of the disease.

Cullen's synopsis is, "burning fever, pain in the head, redness of the face and eyes, intolerance of light and sound, wakefulness, fierce delirium, or low muttering delirium."

This definition may, for aught we know, be a faithful picture of the disease as it occurs in some rare cases though such cases must be confined mostly to hot climates. Only one of the symptoms thus enumerated has been found uniformly present, viz.: "pain in the head." Eberle gives "synocha, fixed and intense throbbing pain in the head, face full and flushed, eyes inflamed, intolerance of light, hearing at first morbidly acute, and at last almost complete deafness, furious delirium from the commencement, and constant wakefulness."

This is a little enlargement upon Cullen's synopsis, but by no means an improvement. It is more objectionable than his from which it was evidently mostly borrowed, making "furious delirium from the commencement" a symptom, whereas Cullen does not state when it occurs, leaving it to be inferred that it may take place at any later period of the disease, which is a fact. Of the many cases in our note book not one was accompanied with "furious delirium from the commencement," although occasional transient mild delirium was present in several from an early stage, and in some was the first symptom to attract attention. Goode's definition is the same with Cullen's except fever, which he drops.

Armstrong's diagnosis is worthy more extended notice from its approximating the appearance of the disease, as it has presented itself to us, much nearer than those we have mentioned. He attempts no synopsis, probably from finding none such of general application.

1st. "Some pain, throbbing, or other uneasiness in the head." He proceeds to say that the pain varies according to the duration and intensity of the disease, is more acute when the membranes alone are inflamed than when the substance of the brain alone is affected, and that it is more distinct towards evening. The latter is almost uniformly true. He farther tells us the pain is increased by the erect position, by light, noise, coughing, and shaking the head. All this is strictly in accordance with our observation, except the increase of pain by light, which we have more rarely observed, and which, when present is not so great as that from noise, coughing, or shaking the head. In later stages, the pain is not so much complained of, *if at all*, in consequence of diminished sensibility, or torpor.

2d. "More or less unnatural dropping of one or both eyelids." Once only have we seen this.

3d. "Cornea more glassy or glaring than natural, while at the same time the intellectual faculties are probably duller." We fancy but little reliance is to be placed in these symptoms.

4th. "Pupils contracted, variable, or dilated." "Variable" best expresses the condition of the pupil. Sometimes in the first stage, it is contracted; in other cases it contracts and dilates alternately, with great rapidity. In later stages it is usually dilated, though not permanently so, unless there is effusion; that is, it will obey the stimulus of light.

5th. "Conjunctiva generally streaked with red." We have noticed this, though not "generally."

6th. "Wakefulness in the first, and heaviness in the last stage." These symptoms occasionally occur, though neither of them is uniformly present. In some cases the heaviness amounts to torpor; in others the patient is merely dull and fretful. When wakefulness is present it is not unfrequently accompanied with great restlessness, as tossing in bed, getting up or otherwise constantly changing posture.

Armstrong makes the mental symptoms—

1st. "Incompetency; an inability to fix the mind on anything or to reason;" and

2d. "Reverie." The last is not unusual; being only occasional, however, in any one case, and generally towards evening.

3d. "Delirium"—but he very correctly adds "delirium seldom occurs in this country [England] before the second, third, or fourth days of the attack." This is precisely our experience in Indiana. Armstrong farther adds—"Delirium does occur, however, in hot countries, at the very outset of the attack." Undoubtedly, and hence the synopsis of Cullen and Eberle, *et id omne genus*, who have copied their descriptions from each other and from tropical writers. He proceeds to enumerate, among other symptoms of the earlier stage, "usual throbbing of the carotid and temporal arteries," increased heat of scalp, face and neck, an irritable stomach or torpid bowels or both. The same remark can be made of these as of his third enumeration of physical symptoms already mentioned.

When a fatal termination is approaching (his second stage) he gives the following as the symptoms, viz.: "Diminution of sensibility." Of one class of cases this is true; the patient becoming more and more heavy until perfectly stupid and insensible. "Pupils first dilated, then immovable." "Patient has a vacant stare or squint." The stare we have often seen, but strabismus is rare in adults. "Pulse first slower then quicker." This change is usually very manifest.

"Subsultus and difficult swallowing." We have never seen the first of these except in cases of long standing, which had assumed a somewhat chronic form, and the second only when a consequence and accompaniment of convulsions. "Convulsions" are likewise mentioned as a symptom of this stage. They are not uncommon in children, but rarely occur in adults, except at a late stage, and indeed in but few cases then.

Mackintosh first gives Cullen's general definition, and adds that after the continuation of these symptoms for a day or two "coma steals on, followed by partial or general convulsions and death." He proceeds: "This case," (a case answering Cullen's definition,) "is the rarest case to be seen in practice," and wonders that Cullen, in drawing out a description of the disease, should have adopted it. In this climate such a case is truly rare, and Cullen's synopsis, so generally adopted and copied, has led to much confusion and malpractice. Mackintosh's description of the disease under the various forms and circumstances under which he has seen it is valuable, as it enables the physician to detect the same morbid affection under entirely dissimilar trains of symptoms.

Doct. Elliotson (Pract. Med.) enumerates as the prominent symptoms, constriction across the forehead, throbbing pain, increased sensibility, *delirium* "*ferox*," pyrexia, full, hard, and accelerated pulse.

Doct. Watson, (Pract. Phys.) and Doct. A. Crawford (Cyc. Pract. Med.) give much the same prominent symptoms. All these gentlemen admit variety in their appearance, but aver the above to be most usually present.

We will now describe the disease as it has repeatedly presented itself to us within the last thirteen years, premising only that its usual period of occurrence is during the months of January, February, and March.

The patient complains of headache, generally *quite severe*, in some part of the frontal region; perhaps confined to one spot, as over an eye, or more diffused, and accompanied with a sense of heaviness or fulness in the head. The pain is increased by coughing, sneezing, or sudden motion (shaking) of the head, and by the erect position. In some cases there is a chilly sensation after the pain has continued a longer or shorter period, followed by its increase. There is very little if any intolerance of light or noise. The nostrils are early found to be unnaturally dry, and the patient complains of their being "stopped up." The appetite not disturbed, though the tongue is slightly coated. The pulse may be somewhat accelerated with other mild feverish symptoms, though as often, perhaps more frequently, the pulse is slower than natural, with increase of fulness. The pain in the head is increased towards evening, becoming sometimes excruciating, with febrile exacerbation. The patient does not sleep well. Towards morning there is a remission of pain and fever, and a disturbed slumber. Hence the disease is not unfrequently mistaken for and treated as a remittent. These symptoms, without any alteration of the pupil, or any delirium, may continue from one to three or four days, the headache rather increasing, but with little change otherwise. The stomach, in some few instances, becomes irritable at an early period of this stage; in others, and the greater number, it is not disturbed at all. After the continuance of the disease thus, for the above mentioned period, if not checked by appropriate treatment, one of two changes manifests itself.

The patient either complains less of pain, becoming drowsy, and the drowsiness gradually increasing to coma; pulse, even if before accelerated, becoming slower; eyes half closed most of the time, and when open staring, but in such manner as to evince want of consciousness; pupil dilated, though still obeying the stimulus of light until a short time prior to the fatal termination, or it may be, until this time, rapidly changeable, alternately contracting and dilating. Unfavorable symptoms increase with more or less rapidity. The coma becomes complete; the pulse again rapid and feeble; the extremities cold; and the patient sinks with or without convulsions.

We said one of two changes occurred—either the above or the headache becomes from bad to worse, until the patient is observed to be delirious—in common parlance, "flighty." The delirium soon becomes furious, with flushed face and wild eyes. The patient struggles incessantly to rise from bed, or to escape from those about him; or he tosses and rolls from side to side of the bed, seldom speaking. These furious muscular exertions continue until he is completely exhausted, and perhaps dies before his last struggle has fairly ceased; or the struggles become weaker, cease, and are followed by coma of short duration, and death.

In some cases (very few) after the continuance of headache as described, delirium of a milder character supervenes, the patient only occasionally evincing decided delirium. He will talk irrelevantly; or perhaps at intervals endeavors to leave his bed or his house without any assigned reason. Next follows coma, or most frequently after this form of delirium, convulsions will precede a fatal termination.

